# Hysteria

**Published:** 1867-07

**Authors:** 


### HYSTERIA.

The following is an extract from the letter of a subscriber and friend in the country:

"I have a case of hysteria, in regard to which I would be pleased to have your opinion, by letter or through the Journal. A difficulty in breathing is the predominant symptom. She is thirty-three years old, has had five children, and has been laboring under her present distress for about three months. We have tried many remedies in her case, but more relief has been afforded by blisters to the cervical spine, and the use of valerianate of iron, than any other means. The dyspnœa is not constant, but partially subsides occasionally for several hours."

Hysteria, named by the ancients from the supposed origin in uterine disturbance, was afterwards thought to be a misnoma. There is little doubt now, however, that this particular kind of nervous disease is usually dependent on organic or functional derangement of the uterus, most generally the former. Under such circumstances, permanent relief need not be expected while the fundamental cause exists. The symptoms characterizing this nervous state are the result of reflex nervous action, and may be allayed temporarily by affording additional vigor to the nerves by the use of "anti-spasmodics," so-called—nervous stimulants, such as oil of amber, assafœtida, &c. Counter irritants to the dorsal spine, where tenderness almost invariably exists, are highly useful in thus relieving for the time the distressing symptoms called hysteria.

In order to prevent the recurrence of the symptoms, treatment directed to the uterus itself must be instituted. When engorgement of the organ exists, manifested by a sense of weight, and slight enlargement of the os and neck, often leading to prolapsus, local depletion, by leeches, or scarification of the pendant portion of the organ should be resorted to.

Mere functional derangement of the womb, on account of general or nervous debility, or an impoverished condition of the blood, must be corrected by such means as the nature of the case would indicate. Such means as are likely to remove these causes act as emenagogue.

Ulceration of the os uteri, and chronic inflammation of the vagina and intra-uterine mucous membrane, manifested by leucorrhœa, generally require astringent and alterative or cauterant local applications to the vaginal mucous membrane, and that within the cavity of the uterus, particularly the cervix. This irritability or sub-acute inflammation of the uterine mucous membrane may exist for years without affording any visible evidences of organic disease except fluor albus. By careful inspection thin glairy mucus may be seen emanating from the os, admonishing the practitioner of the existence of uterine catarrh, and the necessity of extending applications to the interior of the uterus. Fluid preparations thrown into the cavity sometimes, though not always, lead to great suffering and alarm, by inducing what is called uterine colic. Nitrate of silver and other solid local applications, though free from this difficulty, are more difficult of application. Various modes have been suggested, but for the ready and safe application to the membrane beyond that lining the neck, we must look to the rising genius of progressive medicine.

